# What Chicago community organizations needed to implement COVID-19 interventions: lessons learned in 2021

**DOI:** 10.3389/fpubh.2023.1221170

**Published:** 2023-07-10

**Authors:** David A. Moskowitz, Abigail Silva, Yvette Castañeda, Samuel L. Battalio, Madison L. Hartstein, Anne Marie Murphy, Sithembinkosi Ndebele, Matthew Switalski, Sarah Lomahan, Leilani Lacson, Abigail Plum, Emma Canty, Anna Sandoval, Paris Thomas, Marina De Pablo, Bonnie Spring, Molly Martin

**Affiliations:** ^1^Biological Sciences Division, Department of Public Health Sciences, The University of Chicago, Chicago, IL, United States; ^2^Department of Public Health Sciences, Loyola University Chicago, Chicago, IL, United States; ^3^Sinai Urban Health Institute, Chicago, IL, United States; ^4^Department of Preventive Medicine, Northwestern University Feinberg School of Medicine, Chicago, IL, United States; ^5^Equal Hope, Chicago, IL, United States; ^6^Institute for Health Research and Policy, University of Illinois Chicago, Chicago, IL, United States; ^7^University of Chicago Medicine, Chicago, IL, United States

**Keywords:** COVID-19, academic-community partnerships, needs assessment, health equity, community-based participatory research

## Abstract

**Introduction:**

As the COVID-19 pandemic placed a spotlight on the health inequities in the United States, this study aimed to determine the local programmatic needs of community organizations (CO) delivering COVID-19 interventions across Chicago.

**Methods:**

In the summer of 2021, the *Chicagoland CEAL Program* interviewed 34 COs that were providing education, testing, and/or vaccinations in communities experiencing poor COVID-19 outcomes. The interviews were analyzed thematically and organized around logistical challenges and funding/resource needs.

**Results:**

The COs routinely offered testing (50%) or vaccinations (74%), with most (56%) employing some programmatic evaluation. Programs utilizing trusted-messenger systems were deemed most effective, but resource-intensive. CO specific needs clustered around sustaining effective outreach strategies, better CO coordination, wanting comprehensive trainings, improving program evaluation, and promoting services and programs.

**Conclusion:**

The COs reached populations with low-vaccine confidence using trusted messengers to overcome mistrust. However, replenishment of the resources needed to sustain such strategies should be prioritized. Leveraging the *Chicagoland CEAL Program* to help negotiate community organizations’ interorganizational coordination, create training programs, and provide evaluation expertise are deliverable supports that may bolster COVID-19 prevention.

**Policy implications:**

Achieving health justice requires that all institutions of power participate in meaningful community engagement, help build community capacity, and infuse health equity throughout all aspects of the research and program evaluation processes.

## Introduction

The COVID-19 pandemic placed a spotlight on health inequities in the United States. During its first two years, COVID-19 affected 98 million Americans and caused over a million deaths nationwide (1). Data from across the country showed that historically marginalized Black and Latinx communities were experiencing a disproportionate share of infection, hospitalization, and mortality (1). In Chicago, Black and Latinx communities, comprising 60% of the population, experienced more than twice the rate of hospitalizations and mortality as White Chicagoans despite comparable COVID-19 infection rates (2). Additionally, from the start of free and widescale vaccine availability (April 2021), the COVID-19 vaccine disparities across Chicago were equally pronounced, with vaccination uptake among Black and Latinx residents falling far below that of White residents (2). Public, private, academic, social, and not-for-profit institutions across the country mobilized to address the needs of the hardest hit communities (3–5). The National Institutes of Health (NIH) also responded by providing support to community-engaged researchers across the country through the Community Engagement Alliance (CEAL) Against COVID-19 Disparities Program to build upon long-lasting partnerships critical for improving diversity and inclusion in our research and programmatic responses to COVID-19 and future public health crises (6).

In April 2021, the NIH funded the *Chicagoland CEAL Program* (CCP) with the mission to improve COVID-19 vaccination and engagement in quality therapeutic care and trials for low-income Black and Latinx communities in the Chicago metro area. Five academic health centers, one urban research institute, and a non-profit advocacy social service agency partnered in a unique seven-principal investigator structure to lead the CCP. Investigators with a strong history of conducting community-engaged and health equity research from across the metro Chicago area’s leading research institutions and medical centers leveraged their scientific expertise and community partnerships to enhance ongoing and future COVID-19 vaccination, prevention, mitigation, and response efforts. The CCP committed to using a community-based participatory research (CBPR) approach and a health justice framework in its efforts. The CBPR approach involves co-learning about issues of concern, reciprocal transfers of expertise, shared decision-making power, and mutual ownership of research products and processes (7). The health justice framework involves the equitable allocation of power and resources to the communities experiencing the worst health status (8, 9). This means that community partners and researchers have distinct but equally critical roles in priority-setting aimed at improving equitable healthcare and public health systems. The community partners bring a deep and contextualized understanding of their constituencies and their needs, while the CCP team has strong methodological expertise in implementation science and qualitative research methods.

When the CCP was initiated, there was an expectation to gather data on COVID-19 disparities and conduct direct activities to support vaccination and address misinformation. However, the disparities in COVID-19 vaccination across Chicago were well known. Local governments, health systems, service organizations, and funders were closely monitoring the COVID-19 metrics and trying to improve access to, and messaging for, vaccination. Despite good intentions, their work often overlapped, and collaborations did not always occur. Instead of jumping directly into this dynamic, the CCP decided to first conduct a rapid needs assessment using qualitative methods that would identify the available resources and gaps regarding COVID-19 vaccination across Chicago. It sought to determine specifically what community organizations (COs) needed to best serve their communities. The following methods and results describe this needs assessment and the subsequent CCP plan it informed.

## Methods

### Recruitment

The first step was to generate a landscape of COVID-19 vaccination programs and their affiliated COs. During July 2021, CCP staff utilized publicly available information, as well as information from the investigators’ existing relationships and knowledge to create a database of Chicago-based vaccination programs (*N* = 186). This database was not comprehensive as it focused on the mainly Black and Latinx community areas of Chicago and suburban Cook County. Additionally, some organizations were affiliated with multiple programs.

Conducting key informant interviews with all COs was not feasible due to time and resource constraints. Therefore, the investigators categorized COs as high, medium, and low priority for interviewing. High priority COs (*N* = 34) were defined as those (1) serving the most vulnerable communities of color in Chicago (10), (2) having some previous implementation of, or partnership with, at least one COVID-19 vaccination program, and (3) planning to continue or grow their COVID-19 vaccination programs. Focusing on high priority CO needs was deemed the best strategy, given CCP was actively looking to fund only active partners with future vaccination program plans. All COs that were contacted for interviews went on to participate in the CCP needs assessment. English, Chinese, and Spanish-serving COs were included, as were those serving minors and the older adults. Each CO was contacted by the CCP investigators most familiar with their operations and/or leadership. COs identified whom they felt was best suited to participate in the interviews; the CCP team then scheduled the interview session at the convenience of the CO. Verbal consent was obtained at the initiation of the interview and documented by the interviewer [The study was approved by the Chicago Area Institutional Review Board (CHAIRb), protocol 2022-1202]. No repeated interviews were conducted.

The following authors and their research staff conducted the interviews: Moskowitz, Silva, Battalio, Hartstein, Thomas, Murphy, and Martin. Researchers and their staff were gender diverse, with researchers/project-leads serving as the primary interviewers asking the questions. Staff (i.e., research coordinators, research assistants, postdoctoral fellows, graduate students) tended to serve as secondary interviewers: present, but relatively silent during the interviews, taking explicit and elaborate fieldnotes on the responses. All primary interviewers had at least 1 year of previous experience interviewing research participants; moreover, the primary interviewers and their staff vigorously reviewed the interview guide and trained on it. All primary interviewers had some degree of previous relationship with the COs they interviewed. This spanned anywhere from years of familiarity that included conducting previous collaborations to knowing the CO only for several weeks prior to the interview. As mentioned, all informants were consented, so they knew the aims of the research, the goals of the needs assessment, as well as next steps following the interviews. Informants also consented to understanding the roles of the primary interviewers and their staff as being funded by the NIH to improve community engagement surrounding COVID-19 prevention.

### Measures

During July and August 2021, investigators developed an interview guide to capture CO needs, understand experiences with the COVID-19 prevention landscape, and most importantly, help generate operational ideas the CCP could implement quickly with community partners (by December 2021). Interviews covered six categories: (1) CO descriptive information (e.g., populations served, mission); (2) CO COVID-19 vaccination, testing, and/or awareness campaigns/programs; (3) CO program data collection and evaluation techniques; (4) factors that COs perceived impact vaccine uptake and testing; (5) barriers and facilitators encountered in the field with program implementation (e.g., interorganizational cooperation, trusted messengers); and; (6) CO specific programmatic COVID-19 needs, including ways the CCP could meet those needs. Interviews occurred primarily through a video conferencing platform and were audio recorded; most interviews took around 60-min to complete. Transcriptions were made from recordings. Due to time constraints, transcriptions were not reviewed by participants; however, the findings were disseminated back to all participants before peer-review. During or immediately after interviews, the investigators and staff conducting the interviews entered respondent descriptive data (e.g., interviewee job titles, organizational mission statements, geographic service areas, populations served, etc.) and interview notes into a REDCap database.

### Data analysis

Descriptive data were organized into categories. In some instances, the data did not fit cleanly. For example, instead of stating their region of the city, some COs listed zip codes—while others listed the entire city. These responses were reviewed by research staff and fit into categories that best applied. Research staff from multiple institutions (*n* = 6) who had neither been involved in the interview guide creation nor in the delivery of the interviews coded the interview transcript data. Using structural coding (11) and grounded theory techniques (12, 13), coders used *Dedoose* (14) to assign codes that were determined *a priori*. Specifically, staff employed structural coding (11) which allowed them to break the transcribed interviews into sections that aligned with the questions from the interview guide. For example, interview data that stemmed from a question such as “describe your vaccination outreach strategies?” were coded under “outreach strategies”; or “did you collect program-level data?” were coded under “evaluation.” Finally, the staff generated *in-vivo* codes which *did not* stem from the interview guide and were organic to the conversation (e.g., individualized techniques the informants used to convince community members to get vaccinated; unique problems navigating local and state bureaucracies). Coded data were organized by investigators into key themes: (1) support for effective strategies, (2) help coordinating with other COs, (3) providing trainings for CHWS, (4) data collection and intervention evaluation, and (5) service and program promotion.

## Results

### The sample

Table 1 shows the characteristics of the 34 COs interviewed between August and November 2021. Most identified as community-based organizations (41.2%) or healthcare organizations (29.4%) (with 5 out of the 10 identifying as federally qualified health centers). COs primarily served Latinx/Hispanic (55.9%), African American (47.1%), children (<18 years old, 38.2%), and older adult (>60 years old, 32.4%) populations. They also served the community areas designated by the Chicago Department of Public Health as being the “highest need” areas across Chicago (10). These are operationalized by the City of Chicago as Health Equity Zones (15). COs focused on the West Side (29.4%), Near South Side (44.1%), Southwest Side (44.1%), and the Far South Side (44.1%). Those interviewed from the COs included executive directors (18.4%), vice presidents (and similar directorial positions; 20.4%), and others involved in programming (e.g., community health workers, outreach workers, and coordinators; 30.6%). Interviews lasted 40–60 min.

**Table 1:** Characteristics of the informants and informant sites.

	Count	Percentage
**Number of informants interviewed**	49^c^	–
Job position of informant^b^		
Senior leadership	8	16.3
Executive director	9	18.4
Directorial position	10	20.4
Medical related position	7	14.3
Other	15	30.6
**Number of interviews conducted**^a^	34^c^	–
Has a vaccine program		
Yes	25	73.5
No	9	26.5
**Has a data program**		
Yes	19	55.9
No	13	38.2
Not asked	2	5.9
Has a testing program		
Yes	17	50.0
No	17	50.0
Has an awareness program		
Yes	28	82.4
No	6	17.6
Type of organization		
Community-based organization	14	41.2
Healthcare organization	10	29.4
Government organization	2	5.9
Faith-based organization	2	5.9
Health system-based community service institution	2	5.9
Community-focused professional organization	2	5.9
Funding agency, non-profit professional org	2	5.9
Population served^b^		
Older adults (>60)	11	32.4
Children (<18)	13	38.2
Black/African American	16	47.1
Latino or Hispanic	19	55.9
Immigrant	8	23.5
Underserved/low income/medicaid	6	17.6
Other	5	14.7
Geographic reach		
National	1	2.9
Statewide	7	20.6
Chicago, ALL	4	11.8
Chicago, partial	21	61.8
Missing	1	2.9
Locations served (Health Equity Zone = HEZ)^b^		
North Central Chicago (HEZ 1)	0	0.0
Northwest Chicago (HEZ 2)	2	5.9
West Chicago (HEZ 3)	10	29.4
Near South Chicago (HEZ 4)	15	44.1
Southwest Chicago (HEZ 5)	15	44.1
Far South (HEZ 6)	15	44.1
Cook County	7	20.6
Suburbs	5	14.7

a One interview conducted in Spanish.

b Multiple populations could be selected; percent derived by dividing by 34. Other includes Middle Eastern, Asian, undocumented, healthcare professionals, and specific medial problems.

c There were 49 key informants that participated in 34 interviews. During some interviews, more than one key informant from the CO participated in the interview, which provided unique and specific perspectives from within the same organization.

### Previously implemented programs

Most COs participated in, or led, COVID-19 vaccination programs (73.5%) and/or testing programs (50%), but only 55.9% collected some data on their programs. Over 80% of COs had awareness programs, such as social media awareness campaigns. Some provided other support programs such as financial assistance funds for those left unemployed from the pandemic, and health resource navigation aid to link populations with vaccination or testing services. Programs employed strategies that ranged from information campaigns, door-to-door outreach across neighborhoods, incentives, partnering with other organizations/other health programs, and (for those with capacity) a combination approach that incorporated all of these.

### Support for effective strategies

Informants were asked to define “which strategies still work” for achieving vaccination among those hesitant and for keeping attendance at programs/events high. Most (30 of 34 COs, 88.2%) strongly advocated the use of trusted messengers as necessary to overcome community and individual fears or misconceptions. Mostly they operationalized trusted messengers as CHWs. Some CHWs recognized and leveraged their familiarity and unique position with the community:

We’ve been in the community…serving tons of people for years. [Community members] might have come to us for a foreclosure 10 years ago, but—“you know who we are because we saved your house”—and so you trust us. Now that we are offering this vaccine, you still trust us, versus some random doctor’s office that popped out of nowhere.

Other COs described trusted messengers as “extremely effective” but “hard to find” and even “harder to create.” Informants suggested CCP might provide or connect them with additional trusted messengers, such as CHWs.

Many informants cited financial incentives for vaccination as “definitely working to get shots in arms.” Others discussed offering social services as a means to attract vaccine hesitant community members and get them “in the door.” Several explained how program and staff flexibility was essential to build community trust, improve community perceptions of CO reliability and fidelity, and grow CO familiarity. However, they also indicated these approaches required additional resources including financial capital, staff, logistical planning, management, and oversight. Informants punctuated their interviews by suggesting CCP might contribute financial and in-kind resources to relieve such administrative stressors and, as a corollary, help the COs continue their COVID-19 prevention programs.

### Needs regarding improving coordination

While all COs interviewed responded to COVID-19 through some form of programming, 22 (64.7%) acknowledged that organizational barriers—specifically, poor coordination between COs and government agencies, and between other COs—often slowed access to, and diminished the effectiveness of, services or information programs. CO informants blamed late development or inconsistent delivery of programs on confusion and poor communication between city, county, state, and federal health bureaucracies and their organizations. They also described weak linkages between local COs which limited their ability to jointly host programs or promote each other’s initiatives. In one instance, a non-medical CO decided to partner with a local for-profit medical agency to meet their community’s needs because they felt ignored by governmental programs and disassociated from other local health-oriented COs. Unfortunately, several interviewed COs never found such partnerships and explained they “could only provide information campaigns” even though they “wanted to give out the vaccine.” Informants underscored the need for coordination and suggested this as an opportunity for the CCP.

### Needs for COVID-19 CHW trainings

Fifteen of the 34 COs interviewed (44.1%) specifically cited the need for CHW COVID-19 trainings. In addition to up-to-date information on COVID-19, COs stated that CHWs needed better training in motivational interviewing techniques aimed at convincing vaccine-hesitant individuals to get vaccinated. Virtually all COs offering vaccination programs acknowledged that the demand for vaccines and testing had almost completely disappeared by the time of the interviews (Fall of 2021): “at this point, it’s really a lot of convincing people and trying to help overcome these barriers… to receive the vaccine.” Some COs simultaneously admitted that their CHWs were undertrained and underprepared to respond to the specifics surrounding COVID-19 prevention:

We’ve had our own providers talk to them [the CHWs and staff], or trainings have consisted of searching and finding stuff that’s available online from the CDC to have them read. But to have actual experts in the field talk to them [the CHWs and staff], that would be a *really* great resource…. There’s stuff out there for program managers, but for frontline staff, there does not seem to be a whole lot out there and they are the ones who are actually talking to the patients. They need these ‘tricks’ and tools in order to engage with people.

Some of the COs that wanted CHW support suggested actual CHW training content such as instructions on how to increase availability, clarity, and trustworthiness of community-facing educational information, and how to better position their organizations as accessible, trustworthy messengers of educational information. CCP indicated that it could provide such trainings, which COs enthusiastically supported as a great way to improve vaccination reach.

### Needs regarding data collection and program evaluation

Thirteen COs (38.2%) explicitly endorsed the need for better (or any) COVID-19 program data collection and evaluation. Informants indicated that their programs were created and implemented so quickly that in many cases, no evaluation plan was created or conducted. COs said the evaluation was limited because CO staff were “stretched thin” and lacked “data collection tools and expertise” to analyze data. Additionally, some COs reported a lack of coordination with institutional partners who usually do that work for the organization. Some COs understood and acknowledged that the lack of program evaluation made it difficult to know if their programs were effective. Inconsistent or nonexistent evaluation was an unavoidable deficiency in their program designs and one that would continue “without real [outside CO] support” through “borrowing” data analysts or finding “additional funding for evaluation.” COs mentioned a need to evaluate not only program outcomes, but also to conduct process evaluations. One CO said they wished they had the capacity to “accompany…vaccine outreach teams as they are out in the community, [to identify] what kinds of conversations are working and—if they are not—what [messaging] do we need to change?” COs felt the structure and skills of the CCP would be appropriate to manage or fulfill their data collection and evaluation needs.

### Needs for service and program promotion

Many of the COs (44.1%) voiced difficulties in successfully promoting their programs and the services they were offering at events or offices. Informants routinely cited inconsistent or low community event attendance, particularly following the Spring 2021 vaccine surge; and there was palpable exhaustion conveyed to the interviewers by those still conducting vaccine drives. They were running low on effective ways to encourage community participation and suggested several ways CCP might fulfill this need. Eleven COs currently running programs (32.4%) discussed needing an improved social media presence to provide trusted and timely information. Informants suspected their current social media following insufficiently connected with priority communities: “It’s hard to get young Black men or anyone young really to follow us [on Twitter or Instagram], let alone come to an event.” They expressed interest in the CCP potentially connecting them with social media consultants or connecting their presence with other COs to improve their following. In addition to help with social media platforms, a handful of organizations discussed needing connections with traditional communication outlets (e.g., announcements on local TV news, promotional spots on popular radio shows, calls during church services for congregational attendance, and even door-to-door campaigns). Informants said that social media insufficiently reached their priority populations (e.g., the older adults) and were curious if the CCP could link them with media outlets, churches, and CHWs who could be present in the community.

## Discussion

Despite the tremendous pressure to act immediately upon receipt of funding in 2021, the CCP took time to conduct a rapid needs assessment with Chicago-based COs actively offering COVID-19 services in Black and Latinx communities. This effort was essential to ensure that the actions undertaken by the CCP would be meaningful and useful, especially considering changes in vaccination and testing patterns over the second half of 2021. Our interviews identified five areas in need of attention: support for strategies that worked, unreliable interorganizational coordination, inconsistent CHW training, underrealized program evaluation planning and data collection, and inadequate program and event promotion. CO staff were fatigued, and resources were depleted. They reported great interest in an infusion of resources, support, trainings, and coordination. These results led directly to action. Specifically, four plans that were co-developed by CCP institutions and COs were implemented in the field as quickly as November 2021: (1) organizational matchmaking to expand or develop programs, (2) implementation of comprehensive CHW trainings, (3) provision of external (i.e., researcher) data collection and evaluation expertise, and (4) additional program/event promotion strategies layered on top of current CO promotional infrastructures.

The CCP formed a Synergies Core to align existing vaccination and research efforts locally, and to support communication and promotion. This included bringing unintroduced organizations together to provide new COVID-19 program opportunities to underserved communities (e.g., linking vaccine providers with food pantries in South Side neighborhoods). It also included the identification of partnerships for COVID-19 vaccination *across* the Chicago-area outreach and communications platforms. To support these goals, micro and macro approaches were employed. CCP investigators individually brought complementary COs together through weekly video calls to develop or strengthen programs; and broadly, a CCP website and social media presence (Facebook, Instagram, Twitter, and TikTok) were created and immediately began sharing and disseminating information citywide, both to and from partners.

In response to the need for CHW and trusted messenger support, the CCP formalized a CHW Core. The CHW Core team included leaders in CHW workforce development, training, and research. This group, which had already begun developing COVID-19 training materials, launched the first free publicly available CHW COVID-19 training in December 2021. These trainings continued and were modified based on ongoing CO input, participant feedback, and changes in the pandemic (e.g., new variants). The CHW Core also made a CHW learning collaborative available and worked to network local CHWs and CHW supporters.

Two approaches were used to support data collection and evaluation. First, access to population-level data portals and analysis services were made available free of charge to COs. Second, the CCP partnered with 17 COs to conduct formal evaluations of their COVID-19 vaccination programs. In these partnerships, the CCP provided technical expertise and full support for evaluation, as well as some financial support for program implementation.

### Limitations

This needs assessment was not without several limitations. Interviewers focused on COs deemed “high” priority given their community proximity and programmatic readiness. However, it is possible that by failing to interview organizations rated as “low” or “medium” priority, key needs were missed and opportunities to improve COVID-19 programming were overlooked. Drawing on local knowledge from Chicago-based community health experts, such organizations could have elucidated why they were not implementing vaccination programs or why they no longer wanted to continue with COVID-19 interventions. In terms of the interviews themselves, CCP investigators used a structured question guide that may have curtailed or stifled COs’ abilities to generate revelatory conversations on unbeknownst needs. Finally, the extremely fast turnaround time between conducting the needs assessment of Chicago-based COs and providing substantive support to COs may have pushed the CCP to invest in the quickest solutions at the sacrifice of more long-term or comprehensive ideas. These limitations aside, the CCP was careful to implement all programming in concert with, or under the supervision of the COs themselves. The evaluation of CCP-CO relationships is forthcoming (16).

### Public health implications

The COVID-19 pandemic unmasked the extent and depth of structural racism that facilitated observed racial/ethnic inequities in vaccination levels and outcomes (17, 18). On April 8, 2021, the Centers for Disease Control and NIH declared racism to be a serious public health threat and committed a portion of their multi-billion-dollar budget to addressing racism and its harmful sequelae (19, 20). While the magnitude of the pandemic’s harm and the influx of resources placed immense pressure on public health researchers to act fast, advancing health equity requires time and commitment. Achieving health justice requires that all institutions of power participate in meaningful community engagement that keep the community at the helm, help build community capacity, and infuse heath equity throughout all aspects of the research and program evaluation processes (21). The CCP actualized this directive by choosing to conduct a rapid, yet community-based, participatory assessment of the needs of the most marginalized Chicago-area populations to implement responsive strategies (22, 23). The assessment led directly to programming and support services that aligned with local needs. The process also worked to enhance trust with COs, which was a necessary step because of the long history of structural racism in the Chicago area. In 2023, as COs shift their attention away from COVID-19 and refocus on directly addressing the social determinants of health, stakeholders must continue to challenge the public health system to remain engaged and committed, even as emergency funding dissipates. The needs assessment conducted by the CCP offers a pathway for others to follow in the effort to advance community-academic research partnerships toward the shared vision of achieving health justice.

## Data availability statement

The raw data supporting the conclusions of this article will be made available by the authors, without undue reservation.

## Ethics statement

The studies involving human participants were reviewed and approved by Chicago Area Institutional Review Board. The patients/participants provided their verbal informed consent to participate in this study.

## Author contributions

MM, YC, AMM, BS, ASi, and DM: concept and design. All authors: acquisition, analysis, or interpretation of data. YC, DM, MM, and ASi: drafting of the manuscript. SN, MS, SL, LL, AP, and EC: contributed to the results section. DM, MM, and ASi: critical revision of the manuscript for important intellectual content. YC and DM: statistical analysis and supervision. ASa and AP: administrative, technical, or material support. DM: had full access to all the data in the study and takes responsibility for the integrity of the data and the accuracy of the data analysis. All authors contributed to the article and approved the submitted version.

## Funding

The author(s) declare that financial support was received for the research and/or publication of this article. This research was, in part, funded by the National Institutes of Health (NIH) Agreement OT2HL158287 (Martin, Lynch, Margellos-Anast, Murphy, Peek, Silva, and Spring). The views and conclusions contained in this document are those of the authors and should not be interpreted as representing the official policies, either expressed or implied, of the NIH.

## Conflict of interest

AMM, SL, LL, and PT were employed by Equal Hope.

The remaining authors declare that the research was conducted in the absence of any commercial or financial relationships that could be construed as a potential conflict of interest.

## Publisher’s note

All claims expressed in this article are solely those of the authors and do not necessarily represent those of their affiliated organizations, or those of the publisher, the editors and the reviewers. Any product that may be evaluated in this article, or claim that may be made by its manufacturer, is not guaranteed or endorsed by the publisher.
