# Corrigendum: What Chicago community organizations needed to implement COVID-19 interventions: lessons learned in 2021

**DOI:** 10.3389/fpubh.2025.1616454

**Published:** 2025-05-06

**Authors:** David A. Moskowitz, Abigail Silva, Yvette Castañeda, Samuel L. Battalio, Madison L. Hartstein, Anne Marie Murphy, Sithembinkosi Ndebele, Matthew Switalski, Sarah Lomahan, Leilani Lacson, Abigail Plum, Emma Canty, Anna Sandoval, Paris Thomas, Marina De Pablo, Bonnie Spring, Molly Martin

**Affiliations:** ^1^Biological Sciences Division, Department of Public Health Sciences, The University of Chicago, Chicago, IL, United States; ^2^Department of Public Health Sciences, Loyola University Chicago, Chicago, IL, United States; ^3^Sinai Urban Health Institute, Chicago, IL, United States; ^4^Department of Preventive Medicine, Northwestern University Feinberg School of Medicine, Chicago, IL, United States; ^5^Equal Hope, Chicago, IL, United States; ^6^Institute for Health Research and Policy, University of Illinois Chicago, Chicago, IL, United States; ^7^University of Chicago Medicine, Chicago, IL, United States

**Keywords:** COVID-19, academic-community partnerships, needs assessment, health equity, community-based participatory research

In the published article, there was an error in the Funding statement. It originally stated:

This research is supported by a grant from the National Institutes of Health, OT2HL161610 (Martin, Lynch, Margellos-Anast, Murphy, Peek, Silva, and Spring). The content is solely the responsibility of the authors and does not necessarily represent the official views of the National Institutes of Health.

The correct Funding statement appears below.

**Funding**

The author(s) declare that financial support was received for the research and/or publication of this article. This research was, in part, funded by the National Institutes of Health (NIH) Agreement OT2HL158287 (Martin, Lynch, Margellos-Anast, Murphy, Peek, Silva, and Spring). The views and conclusions contained in this document are those of the authors and should not be interpreted as representing the official policies, either expressed or implied, of the NIH.

The authors apologize for this error and state that this does not change the scientific conclusions of the article in any way. The original article has been updated.

